# Developing an educational blueprint for surgical handover curricula: a critical review of the evidence

**DOI:** 10.1007/s10459-025-10410-1

**Published:** 2025-02-01

**Authors:** Anastasija Simiceva, Jessica M. Ryan, Walter Eppich, Dara O. Kavanagh, Deborah A. McNamara, Marie Morris

**Affiliations:** 1 Department of Surgical Affairs, RCSI, 121 St. Stephen’s Green, Dublin, Ireland; 2 RCSI StAR PhD Programme, St. Stephen’s Green, Dublin, Ireland; 3 The Bon Secours Hospital, Glasnevin, Dublin, Ireland; 4 Faculty of Medicine, Dentistry and Health Sciences, University of Melbourne, Melbourne, Australia; 5 Department of Surgery, Tallaght University Hospital, Dublin, Ireland; 6 Office of the President, RCSI, 123 St. Stephen’s Green, Dublin, Ireland; 7 National Clinical Programme in Surgery, RCSI, Dublin, Ireland; 8 Department of Surgery, Beaumont Hospital, Dublin, Ireland

**Keywords:** Handover, Handoff, Sign-out, Surgical education, Surgical training

## Abstract

*Background* Currently no guidelines exist for the development of surgical handover educational curricula. This critical review synthesises the relevant literature to identify best approaches to handover education and develop an evidence-based framework for teaching and assessing surgical handover skills.* Methods* The following resources were critically reviewed by two independent researchers to identify key educational components; (1) all published studies primarily utilising an educational intervention to improve surgical handover up to May 2023, (2) key international guidelines and (3) reviews of all handover interventions published within the last 10 years.* Results* A total of eight comparative studies, two systematic reviews, and four handover guidelines were included. Findings were reported across eight domains; including educational setting, approach, format, content, resources used, assessment, student feedback, and follow-up training. A framework for developing surgical handover curricula was also reported.* Conclusion* The reported educational framework or ‘blueprint’ aims to assist educators across multiple settings to develop evidence-based surgical handover curricula for undergraduate and postgraduate learners. Future studies need to achieve higher Kirkpatrick levels to demonstrate both effectiveness and sustainability of educational interventions, ensuring safer patient care.

**Supplementary Information:**

The online version contains supplementary material available at 10.1007/s10459-025-10410-1.

## Introduction

Handover entails transfer of patient information and care responsibility between healthcare professionals (Abdellatif et al., 2007; Smith et al., 2015). Poor handover leads to adverse patient outcomes (Abdellatif et al., 2007; Horwitz et al., 2008; Lingard et al., 2004), poor patient satisfaction, longer hospital stays (Gordon et al., 2018), and treatment delays (Desmedt et al., 2021). All healthcare professionals, including those in training, must be able to perform this skill effectively (Abdellatif et al., 2007).

The introduction of the Accreditation Council for Graduate Medical Education (ACGME) duty-hour restrictions and the European Working Time Directive (EWTD) have led to reduced shift times, and therefore increased patient handovers. Effective handover requires complex skills and repeated practice to consolidate key competencies (Holt et al., 2020). To achieve this goal, incorporating the teaching of handover skills across the spectrum of clinical practice is essential (Abdellatif et al., 2007). Although some medical educators have emphasised handover skills for students and practising medical staff, educational gaps remain, and teaching and assessment methods are heterogeneous (Gordon et al., 2018).

The most prominent international handover guidelines were published as early as 20 years ago (Australian Medical Association, 2006; Bywaters et al., 2004; RCSE, 2007), but due to the lack of available evidence at that time, cite only one comparative study between them. No handover guideline includes a structured curriculum specifically focused on handover training. There remains a need for universally applicable frameworks to develop discipline-specific handover curricula that are based on current evidence.

This critical review aims to; (1) determine the optimal setting, approach, format, and content for surgical handover education, (2) identify methods of assessment and feedback approaches and (3) provide educators with an evidence-based blueprint for teaching and assessing handover skills.

## Materials and methods

### Study design

Multiple sources of literature were critically reviewed (Kahlke et al., 2023) to develop a curricular framework (‘blueprint’) for surgical handover; including comparative studies, systematic reviews, and handover guidelines. While comparative studies were identified systematically, the inclusion of other sources of information were deliberately selective, rather than exhaustive (Eva, 2008; Kahlke et al., 2023). The author group comprises educational experts in surgery and healthcare communication, and so where gaps in the evidence were identified, expert opinion was used to address these areas.

### Search strategy and study selection (Table 1)

**Table 1:** Information source inclusion and exclusion criteria

Type of information source	Inclusion criteria	Exclusion criteria
1. Comparative studies	Population/setting: All surgical physician learners (undergraduate and postgraduate)	Educational intervention is insufficiently described to replicate
	Intervention: Primarily an educational intervention with the aim of improving surgical handover practice	
	Comparison: Any (e.g., routine handover practice, no intervention received)	
	Outcomes: Any	
2. Systematic reviews	Population: All learners	Reviews published more than 10 years ago
	Intervention: Any interventions aiming to improve healthcare handover (any discipline)	No/minimal educational findings reported
	Comparison/control: Any	Same findings reported in a more recent review (e.g., in a systematic review of systematic reviews)
	Outcomes: Any educational outcomes	
3. Guidelines	Guidelines covering handover in healthcare settings	No educational recommendations reported

This review builds on the methodology reported in a previous systematic review of surgical handover interventions (Ryan et al., 2024a), conducted in accordance with the PRISMA (Page et al., 2021) and AMSTAR (Shea et al., 2017) guidelines. Original, comparative, studies were included if they primarily used an educational intervention to improve surgical handover practice in undergraduate and postgraduate settings. Articles were excluded if the educational intervention was insufficiently described. The previous systematic review excluded studies involving students and newly appointed doctors who had not yet entered clinical practice; these excluded studies, and any relevant included studies (Ryan et al., 2024a), were reassessed for the current critical review. The described search strategy (Ryan et al., 2024a) was updated to May 2023. With the help of an information specialist, a new education-specific search was also carried out on the PubMed, Ovid Medline, MedEd and Web of Science databases (full search terms are provided in Online Resource 1). The original Endnote (X20) library (Ryan et al., 2024a) was also searched for systematic reviews of all healthcare handover interventions from the last 10 years. Additionally, healthcare handover guidelines which reported educational recommendations were included (Table 1). Study screening and selection were carried out as previously described (Ryan et al., 2024a).

### Data extraction

Templates were created using Microsoft Excel and Word (^©^2022 Microsoft). A subset of papers was allocated to two reviewers (JR and AS) for independent primary data extraction, with subsequent validation of all papers by the second reviewer. Any discrepancies were resolved by consensus with the wider research team. For each comparative study, data on the study design, learner characteristics, sample size, format and content of education, outcomes, and Kirkpatrick level (Kirkpatrick & Kirkpatrick, 2006) were extracted. Where Kirkpatrick level was not reported in the manuscript, the authors inferred this information from the outcomes reported. For systematic reviews, the aims, search limits, total included studies, target learners, main educational findings, and highest study Kirkpatrick level were extracted. Only educational recommendations included in the guidelines were extracted.

### Data synthesis & quality assessment

Extracted data were reported in the following categories; educational setting, approach and format, content, resources utilised, assessment of training effectiveness, student feedback, and follow-up training and assessment.

Interventional studies were assessed according to Kirkpatrick levels of educational impact (Kirkpatrick & Kirkpatrick, 2006).

## Results

### Search results

**Fig. 1:**
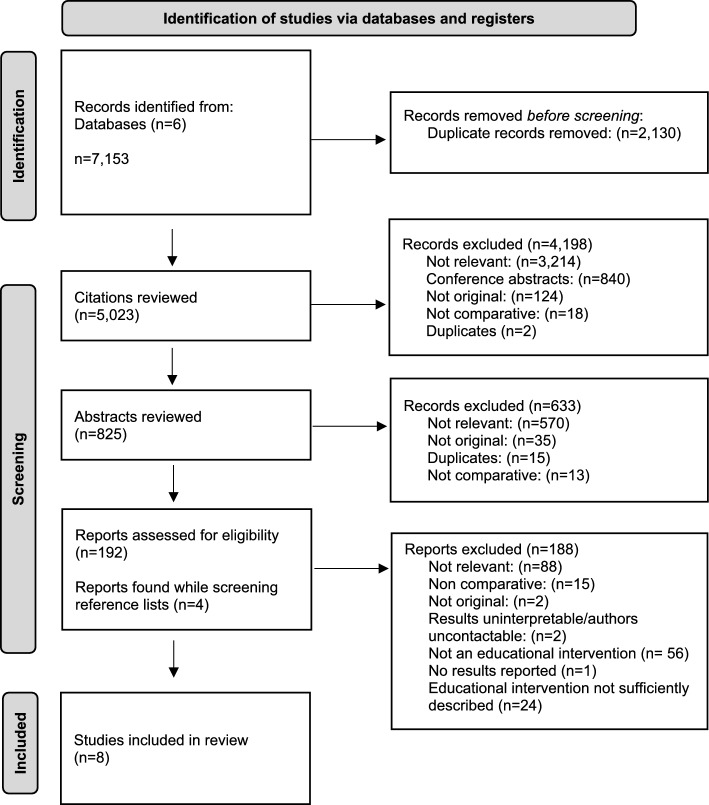
PRISMA Flow Diagram

In total, eight comparative studies were identified for inclusion in this review (Bevilacqua et al., 2020; Britt et al., 2015; Gaffney et al., 2016; Holt et al., 2020; Kulaylat et al., 2023; Ottinger et al., 2017; Smith et al., 2015; Telem et al., 2011). The 7,153 citations retrieved through database searches were screened and a full-text review was performed on 192 papers (Fig. 1). After reviewing the full texts for 25 handover reviews identified during the initial search, data were extracted from five papers. From this, a total of two systematic reviews were included (Online Resource 2) (Desmedt et al., 2021; Gordon et al., 2018). Data were extracted from six handover guidelines (Online Resource 3); however, only four of these included specific educational recommendations (Abdellatif et al., 2007; Australian Medical Association, 2006; Canadian Association of General Surgeons., 2018; National Clinical Effectiveness Committee, 2015), and so the rest were excluded.

### Study characteristics

Of the eight included comparative studies (Bevilacqua et al., 2020; Britt et al., 2015; Gaffney et al., 2016; Holt et al., 2020; Kulaylat et al., 2023; Ottinger et al., 2017; Smith et al., 2015; Telem et al., 2011), five adopted a pre-/post-intervention cohort design (Bevilacqua et al., 2020; Gaffney et al., 2016; Holt et al., 2020; Ottinger et al., 2017; Smith et al., 2015), two were case–control studies (Kulaylat et al., 2023; Telem et al., 2011), and one was a randomised controlled trial (Britt et al., 2015) (Table 2). A total of 303 students, interns, and residents were included across the studies, the majority of which (n = 7) were carried out in the USA (Bevilacqua et al., 2020; Britt et al., 2015; Gaffney et al., 2016; Kulaylat et al., 2023; Ottinger et al., 2017; Smith et al., 2015; Telem et al., 2011).

**Table 2:** Comparative study characteristics

Author, year	Country	Learners	Sample size	Study design	Format & content of education	Summary of results	Kirkpatrick level
Bevilacqua et al., 2020	USA	4th year students matched to surgical specialties	12	Pre-/post-intervention cohort	1-h module entitled “Practice handoffs” Incorporated into a 2-week surgical boot camp	Improvement in handoff performance according to an ACS standardised grading form (*p* < 0.01)	2
Britt et al., (2015)	USA	Surgical interns	10	RCT	Interactive lecture: structure and content of handover based on the handover assessment tool designed by the research team. Followed by simulated handover practice with feedback	Improvement in overall handover performance according to the handover assessment tool in the trained group (*p* < 0.001)	2
Gaffney et al., (2016)	USA	Incoming residents from multiple specialties including surgery	84	Pre-/post-intervention cohort	Online handover training module including 4-min video highlighting pitfalls, 15-min didactic screencast, and an MCQ assessment. Followed by in-person simulated handover practice with feedback	Increase in self-reported preparedness to conduct a handover (likert scale survey) after the intervention (*p* < 0.001)	1
Holt et al., (2020)	UK	3rd and 4th-year students in surgery and medicine	41	Pre-/post-intervention cohort	2.5 h interactive handover workshop. Including simulated practice with feedback, group discussion, & video examples of handover	Improvement in learner self-reported confidence in delivering handovers (survey; *p* < 0.0001) Significant improvement in handover performance on OSCE assessment using the CHAT tool (10 of 12 domains; *p* < 0.05)	2
Kulaylat et al., 2023	USA	3rd year medical students rotating through surgical clerkships	39	Case control study	90-min handover training session: Including didactic lecture, group discussion, videos of good and bad handover, and guided practice both at the beginning, and end of the session to synthesise learning	No difference in knowledge (MCQ test), confidence, or anxiety (likert scale survey). Those who received the intervention were more likely to have actively participated in a handover during their clerkship after the intervention (*p* < 0.05)	3
Ottinger et al., (2017)	USA	Trauma surgical residents	NR	Pre-/post-intervention cohort	Education session: Important handover and communication skills, outline of “Trauma morning report faculty evaluation card”, & open assessment of residents presentation skills	Improvement in inclusion of handover information and communication skills of the residents according to the evaluation tool (*p* < 0.01)	2
Smith et al., (2015)	USA	Medical students matched to multiple specialties including surgery	59	Pre-/post-intervention cohort	3-h handover workshop: Including didactic lectures, role-play activities, and group discussion	Improvement in attitudes and confidence towards handover (*p* < 0.01). Improved handover knowledge and ability to write a verbal handover dialogue (*p* < 0.001; participant survey and MCQs). Performance had significantly disimproved on follow-up testing several months later, before commencing residency (training did not coincide with start of clinical duties)	2
Telem et al., (2011)	USA	Surgical residents	58	Case control study	2.5-h handover curriculum: Including videos of good and bad handover, group discussion, role-play scenarios, and didactic lecture on SBAR	Reduction in erroneous order entries in the intervention group (p = 0.003). No change in patient sentinel events	4

^*^CHAT Clinical handover assessment tool, *MCQ* Multiple choice question, *OSCE* Objective structured clinical examination, *ISBAR* Identify Situation Background Assessment Recommendation, *ACS* American College of Surgeons, *SBAR* Situation Background Assessment Recommendation, *RCT* Randomised Control Trial, *NR* not reported

### Educational findings

**Fig. 2:**
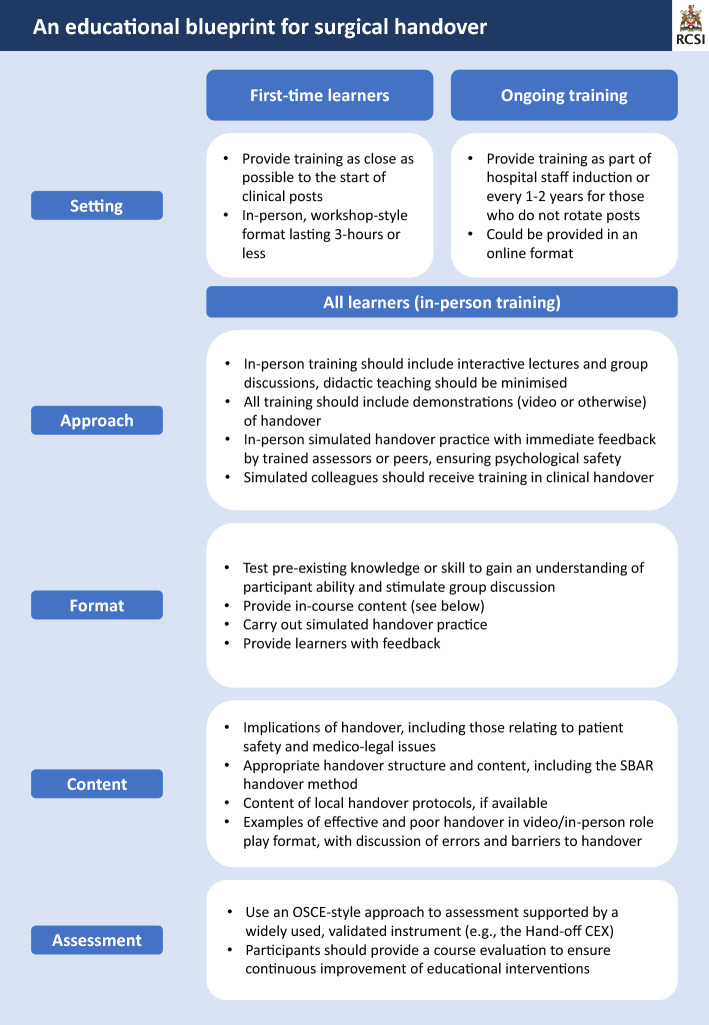
An Educational Blueprint for Surgical Handover

The following results have been used to develop a curricular framework (Fig. 2) to assist educators in developing evidence-based surgical handover curricula.

#### Educational setting

Handover training is recommended as part of in-hospital staff induction (Abdellatif et al., 2007); however, most surgical literature focuses on educational interventions for undergraduate settings (Bevilacqua et al., 2020; Gaffney et al., 2016; Holt et al., 2020; Kulaylat et al., 2023; Smith et al., 2015). Previous training has been delivered in a workshop-style format (Holt et al., 2020; Smith et al., 2015) and incorporating handover training into ‘surgical boot camps’ improves handover performance (Bevilacqua et al., 2020). No consensus exists on optimal training duration, with in-person workshops lasting a median of 120 (40–180) minutes (Bevilacqua et al., 2020; Holt et al., 2020; Kulaylat et al., 2023; Smith et al., 2015; Telem et al., 2011). Online training lacks the benefit of in-person handover practice and has been inadequately tested with only one prior study using this method (Gaffney et al., 2016). The authors propose that it would have value in the form of ‘refresher’ courses for staff who have previously undergone in-person training, for example during hospital induction.

#### Educational approach and format

Most studies utilised combined approaches, including didactic (Gaffney et al., 2016; Kulaylat et al., 2023; Ottinger et al., 2017; Smith et al., 2015; Telem et al., 2011) and interactive lectures (Britt et al., 2015), group discussions (Gaffney et al., 2016; Holt et al., 2020; Kulaylat et al., 2023; Telem et al., 2011), video demonstrations of handover (Britt et al., 2015; Gaffney et al., 2016; Holt et al., 2020; Kulaylat et al., 2023), and simulated handover practice with (Smith et al., 2015; Telem et al., 2011) or without (Britt et al., 2015; Gaffney et al., 2016; Holt et al., 2020) feedback. Students prefer simulation-based education and role-play over didactic sessions (Desmedt et al., 2021). Little information is provided on the recommended format of simulation (National Clinical Effectiveness Committee 2019); however, simulated colleagues do receive training before participating in handover (Britt et al., 2015; Gaffney et al., 2016; Holt et al., 2020).

There are two broad categories for structuring workshops: (1) information sharing and (2) opportunities for active practice, and the most utilised formats include (Gordon et al., 2018);

1. Practice handover, receive teaching, practice again, receive feedback
2. Receive teaching, practice handover, receive feedback
3. Test pre-existing knowledge, receive teaching, practice, receive feedback

#### Educational content

International guidelines recommend that optimal handover content (Canadian Association of General Surgeons., 2018), technique (Abdellatif et al., 2007), medico-legal issues and local protocols (Australian Medical Association, 2006) are covered. Interventional studies include; the importance of handover (Holt et al., 2020; Smith et al., 2015), appropriate structure and content (Britt et al., 2015; Holt et al., 2020; Ottinger et al., 2017; Smith et al., 2015), clinical scenarios (Holt et al., 2020), video examples of good and bad handover (Gaffney et al., 2016; Kulaylat et al., 2023; Telem et al., 2011), and discussion of common errors (Smith et al., 2015) and barriers to handover (Holt et al., 2020). Students rate video examples of handover highly (Holt et al., 2020).

Various mnemonics to teach handover methods (Holt et al., 2020; Smith et al., 2015; Telem et al., 2011) have also been included in previous course content. SBAR (Situation, Background, Assessment, Recommendation), is the most common method used internationally (Desmedt et al., 2021). Pre-existing (Bevilacqua et al., 2020; Holt et al., 2020), or newly designed (Britt et al., 2015; Ottinger et al., 2017), assessment tools were also used to guide handover curricula. For example, Britt et al., designed a Global Handoff Rating Scale for their randomised controlled trial which structured the content of an interactive lecture (Britt et al., 2015).

#### Resources used

Regarding resources used, educational interventions required; staffing/faculty (Bevilacqua et al., 2020; Britt et al., 2015; Gaffney et al., 2016; Holt et al., 2020; Ottinger et al., 2017; Smith et al., 2015; Telem et al., 2011), standardised colleagues (Britt et al., 2015), video (Gaffney et al., 2016; Holt et al., 2020; Smith et al., 2015; Telem et al., 2011) and role play scenario development (Bevilacqua et al., 2020; Britt et al., 2015; Holt et al., 2020; Smith et al., 2015; Telem et al., 2011), and administration (printing, documentation) costs (Holt et al., 2020). One study highlighted the cost effectiveness of written scenarios for both training and assessing handover skill (Britt et al., 2015). An online training module was also used prior to in-person practice (Gaffney et al., 2016). No studies carried out a formal cost analysis of the intervention used.

#### Assessment of training effectiveness

In terms of Kirkpatrick levels of educational impact (Kirkpatrick & Kirkpatrick, 2006) only one interventional study achieved level IV, demonstrating a significant reduction in erroneous order entries 30-days post-handover training (Telem et al., 2011). Two further studies reported level II outcomes (Holt et al., 2020; Smith et al., 2015), with one study achieving level III (Holt et al., 2020).

Some studies have used self-reported measures to evaluate outcomes, with educational interventions significantly improving confidence, preparedness, and ability to conduct verbal handover (Gaffney et al., 2016; Holt et al., 2020; Smith et al., 2015). Undergraduate students also reported that they were more likely to participate in written or verbal handover after education (Kulaylat et al., 2023). Other studies used tests of written knowledge, including Multiple Choice Questions and tests of written handover dialogue (Kulaylat et al., 2023; Smith et al., 2015). Guidelines recommend verbal and face-to-face handover where possible (Abdellatif et al., 2007; National Clinical Effectiveness Committee, 2015), therefore, the authors assert that assessment of simulated practice rather than written handover dialogue may be a better use of resources.

Multiple assessment methods have been used to evaluate surgical handover performance during simulation, including, the ‘Handoff CEX’ tool (Gaffney et al., 2016), Objective Structured Clinical Examination (OSCE) station using a modified version of the ‘Clinical Handover Assessment Tool’ (CHAT) (Holt et al., 2020), and a ‘Handover Global Assessment Scale’ (Britt et al., 2015). Several of these lack rigorous validity and reliability testing (Britt et al., 2015; Ottinger et al., 2017); however, the most widely used assessment tool is the ‘Hand-off CEX’ (Desmedt et al., 2021).

#### Student feedback

Some learners received immediate feedback based on their handover performance (Britt et al., 2015; Gaffney et al., 2016; Holt et al., 2020), which was provided based on a rating scale and/or check list (Britt et al., 2015; Gaffney et al., 2016) by trained evaluators and peers (Gaffney et al., 2016; Smith et al., 2015). Providing feedback after handover practice is well-received by students (Holt et al., 2020). Learners found handover curricula to be beneficial (Gaffney et al., 2016; Holt et al., 2020), helpful (Gaffney et al., 2016; Telem et al., 2011), and representative of common communication failures (Telem et al., 2011). Many would also recommend the educational programme to others (Gaffney et al., 2016; Telem et al., 2011).

#### Follow-up training and assessment

No interventional studies provided follow-up training after the initial workshop and follow-up assessment for undergraduate learners demonstrated no sustained improvement in knowledge (Kulaylat et al., 2023; Smith et al., 2015). Smith et al. found that while scores improved immediately following their student workshop, they lost skill gains on follow-up testing (Smith et al., 2015). Notably, these participants had not yet begun residency and lacked opportunities to practice in a clinical context, amplifying skill decay. Whereas follow-up testing in postgraduate learners (i.e. those already working in clinical roles) demonstrated sustained improvement in handover skills (Ottinger et al., 2017).

## Discussion

This critical review synthesises best available evidence in surgical handover education from relevant comparative studies, systematic reviews, and international guidelines, offering educators a model adaptable to undergraduate, postgraduate, and low-resource surgical training environments. The findings highlight the importance of interactive, simulation-based approaches to handover training, individual immediate feedback on handover performance, and the opportunity for deliberate repeated practice. Guidelines for the optimal educational setting, approach, format, content, and assessment have been described.

Handover competency is an under-taught key professional requirement (General Medical Council, 2019; Irish Medical Council, 2024) which poses a risk to patient safety. One-third of surgical residents feel that handover practices in their institution are unsafe and 5% report a recent occurrence of moderate patient harm as a result, yet only 11% have ever received any formal training (Ryan et al., 2024b). Improved handover significantly reduces patient length of stay (Ryan et al., 2024a), medical errors, and preventable adverse events (Starmer et al., 2014). Additionally, introducing handover teaching at an early stage promotes confidence in medical students (Holt et al., 2020; Smith et al., 2015) and increases their likelihood of engaging in handover while on clinical attachments (Kulaylat et al., 2023). The proposed framework should assist surgical educators in providing evidence-based handover training, encouraging safer handover practices and reducing risk to patients.

Future research should focus on evaluating and improving knowledge retention over time and the sustainability of interventions. Training should be scheduled as close as possible to the commencement of clinical duties to allow for regular reinforcement, consolidation of learning, and avoidance of skill decay (Kulaylat et al., 2023; Ottinger et al., 2017; Smith et al., 2015). The use of simulation should be further explored as it is one of the most effective ways of teaching complex skills (Chernikova et al., 2020); however, methods must be accurately reported so that they are replicable. The authors also recommend that educational programs aim to accomplish and report outcomes at levels of III or above, to demonstrate the use of skills in practice and a positive impact on patient outcomes (Kirkpatrick & Kirkpatrick, 2006). Online training also warrants further exploration, as it could increase remote training, limiting staff time away from clinical activities. Finally, an analysis of the cost-effectiveness and resource requirements of training programmes would also provide valuable insights, particularly to support implementation in low-resource healthcare settings.

This review was limited by the quality of the evidence available, with only one study reporting outcomes at Kirkpatrick level IV, limiting assessment of the impact of training on patient outcomes. This is a common theme across interventional studies in surgical handover (Ryan et al., 2024a), and the area would benefit greatly from a core outcome set to standardise reporting of outcomes and increase comparability between studies (Ryan et al., 2024c).

## Conclusions

The best available evidence in this area has been critically reviewed and synthesised to develop a framework to assist educators in developing evidence-based surgical handover curricula. However, international guidance on surgical handover education remains limited, with most interventional studies reporting level II Kirkpatrick outcomes. Future studies should aim to achieve higher Kirkpatrick levels to demonstrate both effectiveness and sustainability of educational interventions, ensuring safer patient care.

## Supplementary Information

Below is the link to the electronic supplementary material.

Supplementary file1 (XLSX 17 KB)

Supplementary file2 (PDF 809 KB)

Supplementary file3 (PDF 33 KB)

## Data Availability

No datasets were generated or analysed during the current study.
