# Supplementary material for: Developing an educational blueprint for surgical handover curricula: a critical review of the evidence
Source: Adv Health Sci Educ Theory Pract. 2025 Feb 1;30(5):1693–707. doi: 10.1007/s10459-025-10410-1 (PMC12572099; doi:10.1007/s10459-025-10410-1)
Supplement: Supplementary file 2 — Supplementary file2 (PDF 809 KB) [file 10459_2025_10410_MOESM2_ESM.pdf]

Developing an educational blueprint for surgical handover curricula: A critical review of the evidence

Advances in Health Sciences Education

Anastasija Simiceva*[a] & Jessica M Ryan*,[a,b,c] Walter Eppich,[d] Dara O Kavanagh,[a,e] Deborah A McNamara MD,[f,g,h] Marie Morris [a]

***Joint first authorship**

Author institutions

[a] RCSI Department of Surgical Affairs, 121 St. Stephen's Green, Dublin

[b] RCSI StAR PhD programme, St. Stephen's Green, Dublin, Ireland

[c] The Bon Secours Hospital, Glasnevin, Dublin, Ireland

[d] Faculty of Medicine, Dentistry and Health Sciences, University of Melbourne, Melbourne, Australia

[e] Department of Surgery, Tallaght University Hospital, Dublin, Ireland

[f] Office of the President, RCSI, 123 St. Stephen's Green, Dublin, Ireland

[g] National Clinical Programme in Surgery, RCSI, Dublin, Ireland

[h] Department of Surgery, Beaumont Hospital, Dublin, Ireland

Corresponding author

Jessica Ryan

jessicaryan@rcsi.com

Online Resource 2. Details of included and excluded handover systematic reviews

Author, year	Journal	Aim of review	Search limits	Total studies	Target learners	Highest Kirkpatrick level	Decision
Masterson 2013[1]	Can Med Ed J	To systematically review the literature regarding education models available to teach handover skills to healthcare professionals	1990 - 2009	12	Residents, interns, nurses, paramedics, medical students, peri-operative specialists	3	Exclude<br><br>**Included in Desmedt 2020**
Davis 2015[2]	J Grad Med Ed	To evaluate current evidence on the effectiveness of electronic solutions used to support shift-to-shift handovers	2000 - 2014	37	Residents, nurses, attendings, fellows, 'mid-level providers'	NR	Exclude<br><br>**Minimal educational conclusions**
Pucher 2015[3]	Surgery	To review evidence regarding handover interventions in surgery and to assess compliance with Joint Commission guidelines	Up to Dec 2013	19	NR – all types of perioperative handovers included	NR	Exclude<br><br>**Included in Desmedt, 2020**
Gordon 2018[4]	Academic Medicine	To systematically review the latest evidence regarding handover education, to describe the features of the reported interventions, and to determine whether the interventions are effective and how they function	2010 -2016	18	Medical students, residents, attending physicians, and nurses	4	Include
Desmedt 2020[5]	Int J Qual Health Care	To systematically review all available systematic reviews on clinical handover	2009 -2018	19		NR	Include

References

1. Masterson MF, Gill RS, Turner SR, Shrichand P, Giuliani M. A systematic review of educational resources for teaching patient handover skills to resident physicians and other healthcare professionals. *Can Med Educ J*. 2013;4(1):e96-e110.
2. Davis J, Riesenberg LA, Mardis M, et al. Evaluating Outcomes of Electronic Tools Supporting Physician Shift-to-Shift Handoffs: A Systematic Review. *J Grad Med Educ*. Jun 2015;7(2):174-80. doi:10.4300/jgme-d-14-00205.1
3. Pucher PH, Johnston MJ, Aggarwal R, Arora S, Darzi A. Effectiveness of interventions to improve patient handover in surgery: A systematic review. *Surgery*. Jul 2015;158(1):85-95. doi:10.1016/j.surg.2015.02.017
4. Gordon M, Hill E, Stojan JN, Daniel M. Educational Interventions to Improve Handover in Health Care: An Updated Systematic Review. *Acad Med*. Aug 2018;93(8):1234-1244. doi:10.1097/acm.0000000000002236
5. Desmedt M, Ulenaers D, Grosemans J, Hellings J, Bergs J. Clinical handover and handoff in healthcare: a systematic review of systematic reviews. *International Journal for Quality in Health Care*. 2021;33(1):mzaa170.
