# Supplementary material for: Developing an educational blueprint for surgical handover curricula: a critical review of the evidence
Source: Adv Health Sci Educ Theory Pract. 2025 Feb 1;30(5):1693–707. doi: 10.1007/s10459-025-10410-1 (PMC12572099; doi:10.1007/s10459-025-10410-1)
Supplement: Supplementary file 3 — Supplementary file3 (PDF 33 KB) [file 10459_2025_10410_MOESM3_ESM.pdf]

Developing an educational blueprint for surgical handover curricula: A critical review of the evidence

Advances in Health Sciences Education

Anastasija Simiceva*[a] & Jessica M Ryan*,[a,b,c] Walter Eppich,[d] Dara O Kavanagh,[a,e] Deborah A McNamara MD,[f,g,h] Marie Morris [a]

***Joint first authorship**

Author institutions

[a] RCSI Department of Surgical Affairs, 121 St. Stephen's Green, Dublin

[b] RCSI StAR PhD programme, St. Stephen's Green, Dublin, Ireland

[c] The Bon Secours Hospital, Glasnevin, Dublin, Ireland

[d] Faculty of Medicine, Dentistry and Health Sciences, University of Melbourne, Melbourne, Australia

[e] Department of Surgery, Tallaght University Hospital, Dublin, Ireland

[f] Office of the President, RCSI, 123 St. Stephen's Green, Dublin, Ireland

[g] National Clinical Programme in Surgery, RCSI, Dublin, Ireland

[h] Department of Surgery, Beaumont Hospital, Dublin, Ireland

Corresponding author

Jessica Ryan

jessicaryan@rcsi.com

Online Resource 3. Summary of handover guideline educational recommendations

Recommendation category	Recommendation	AMA[1]	WHO/ JCI[2]	CAN[3]	IRE[4]
Provision of education	Validated education and training should be provided				X
	Ensure protected time for education				X
	Education should be mandatory				X
	Ensure ongoing in-service education				X
	Interdisciplinary attendance				X
Educational setting	Incorporate training into staff orientation/induction	X	X		X
	Utilise workshops				X
Educational approach	Utilise simulation-based education				X
	Human factors-oriented approach				X
	Promote staff confidence				X
	Encourage mutual respect between HCW and patients				X
	Encourage questioning				X
Educational content	Cover local handover protocols	X			
	Emphasise the importance of two-way communication				X
	Emphasise impact of handover on patient safety				X
	Promote a culture of openness				X
	Cover handover content & technique	X	X	X	
	Cover the importance of clinical notes with examples of good and bad practice	X			
	Include medico-legal issues associated with handover, discharge, and documentation with case studies	X			
	Cover the use of local handover tools	X			

*AMA, Australian Medical Association; WHO, World Health Organisation; JCI, Joint Commission International; CAN, Canada; IRE, Ireland

References

1. Australian Medical Association. Safe handover: safe patients. Guidance on clinical handover for clinicians and managers. Canberra: AMA, 2006.

2. Abdellatif A, Bagian JP, Barajas ER, et al. Communication during patient hand-overs: patient safety solutions, volume 1, solution 3, May 2007. *Joint Commission Journal on Quality and Patient Safety*. 2007;33(7):439-442.

3. Committee on Acute Care Surgery, Canadian Association of General Surgeons. Clinical Practice Guideline: Dynamic Practice Guidelines for Emergency General Surgery. 2018. Accessed March 20 2023. https://cags-accg.ca/wp-content/uploads/2018/11/ACS-Handbook-CPG-Ch-1-Rounding-and-Handover.pdf

4. National Clinical Effectiveness Committee. Communication (Clinical Handover) in Acute and Children's Hospital Services, National Clinical Guideline No. 11. 2015. Available from: https://assets.gov.ie/11589/774c4bb699144120946a091b481f2334.pdf
